# Single arc volumetric-modulated arc therapy is sufficient for nasopharyngeal carcinoma: a dosimetric comparison with dual arc VMAT and dynamic MLC and step-and-shoot intensity-modulated radiotherapy

**DOI:** 10.1186/1748-717X-8-237

**Published:** 2013-10-14

**Authors:** Zhong-Hua Ning, Jin-Ming Mu, Jian-Xue Jin, Xiao-Dong Li, Qi-Lin Li, Wen-Dong Gu, Jin Huang, Yang Han, Hong-Lei Pei

**Affiliations:** 1 Department of Radiation Oncology, The Third Affiliated Hospital, Soochow University, 185 Juqian Road, Changzhou 213003, China; 2 Department of Radiation Physics, Elekta China Co. Ltd, 27 North Fourth Ring Mid Road, Chaoyang District, Beijing 100101, China; 3 Department of Oncology, The Third Affiliated Hospital, Soochow University, 185 Juqian Road, Changzhou 213003, China

**Keywords:** Radiotherapy, Volumetric modulated arc therapy, Radiotherapy, Intensity modulated, Nasopharyngeal carcinoma, Plan evaluation, Dosimetry

## Abstract

**Background:**

The performance of single arc VMAT (VMAT1) for nasopharyngeal carcinoma (NPC) on the Axesse linac has not been well described in previous studies. The purpose of this study is to assess the feasibility of VMAT1 for NPC by comparing the dosimetry, delivery efficiency, and accuracy with dual arc VMAT (VMAT2), dynamic MLC intensity-modulated radiotherapy (dIMRT), and step-and-shoot intensity-modulated radiotherapy (ssIMRT).

**Methods:**

Twenty consecutive patients with non-metastatic NPC were selected to be planned with VMAT1, VMAT2, dIMRT and ssIMRT using Monaco 3.2 TPS on the Axesse™ linear accelerator. Three planning target volumes (PTVs), contoured as high risk, moderate risk and low risk regions, were set to receive median absorbed-dose (D_50%_) of 72.6 Gy, 63.6 Gy and 54 Gy, respectively. The Homogeneity Index (HI), Conformity Index (CI), Dose Volume Histograms (DVHs), delivery efficiency and accuracy were all evaluated.

**Results:**

Mean HI of PTV_72.6_ is better with VMAT1(0.07) and VMAT2(0.07) than dIMRT(0.09) and ssIMRT(0.09). Mean HI of PTV_63.6_ is better with VMAT1(0.21) and VMAT2(0.21) than dIMRT and ssIMRT. Mean CI of PTV_72.6_ is also better with VMAT1(0.57) and VMAT2(0.57) than dIMRT(0.49) and ssIMRT(0.5). Mean CI of PTV_63.6_ is better with VMAT1(0.76) and VMAT2(0.76) than dIMRT(0.73) and ssIMRT(0.73). VMAT had significantly improved homogeneity and conformity compared with IMRT. There was no significant difference between VMAT1 and VMAT2 in PTV coverage. Dose to normal tissues was acceptable for all four plan groups. VMAT1 and VMAT2 showed no significant difference in normal tissue sparring, whereas the mean dose of the parotid gland of dIMRT was significantly reduced compared to VMAT1 and VMAT2. The mean delivery time for VMAT1, VMAT2, dIMRT and ssIMRT was 2.7 min, 3.9 min, 5.7 min and 14.1 min, respectively. VMAT1 reduced the average delivery time by 29.8%, 51.1% and 80.8% compared with VMAT2, dIMRT and ssIMRT, respectively. VMAT and IMRT could all be delivered accurately based on our quality assurance standards.

**Conclusions:**

In the treatment of NPC using the Axesse™ linear accelerator, single arc VMAT has shown superiority to double arc VMAT, dIMRT and ssIMRT in delivery efficiency, without compromise to the PTV coverage. However, there is still room for improvement in terms of OAR sparing.

## Background

Nasopharyngeal carcinoma (NPC) is endemic, especially in southern China. Despite the rapid advancement in chemotherapy and molecular targeted therapy, radiotherapy is still the treatment of choice for NPC. Intensity-modulated radiation therapy (IMRT), the most commonly used in the treatment of NPC, has improved local control and 5-year survival rates with significantly lower radiation-induced toxicities than two-dimensional radiotherapy [1,2]. However, the prolonged delivery time of IMRT poses two disadvantages. Firstly, the prolonged delivery time can decrease efficiency and increase intrafraction uncertainty of target volume localization and dosimetry [3,4]. Secondly, prolonging the fraction time will spare tissues with a fast DNA repair and might decrease tumor cell killing [5-7].

Volumetric-modulated arc therapy (VMAT) can generate precise conformal dose distribution through rotational delivery accompanied by variability of the Multileaf Collimator (MLC) position, dose rate and gantry rotation velocity [8,9]. Compared to IMRT, VMAT can improve the dose distribution, reduce the dose to normal tissues and shorten the delivery time [10]. It has been reported that dual arc VMAT for the treatment of NPC on a Synergy™ (Elekta AB, Stockholm, Sweden) linear accelerator (linac) produced superior results in terms of Planning Target Volume (PTV) coverage and Organs at Risk (OARs) sparing, but was slightly less efficient than single arc VMAT [11]. Compared with Synergy™ linac, Axesse™ linac (Elekta AB, Stockholm, Sweden) is equipped with the improved Integrity™ treatment control system and the newly designed Agility™ head. This paper will address the question whether single arc VMAT, with aforementioned technical improvements, is adequate for NPC with no compromise PTV coverage and OARs sparing.

## Materials and methods

### Patient selection and contouring

This study was approved by ethical committee of Soochow University. 20 consecutive non-metastatic NPC patients were enrolled in this study between 2011 and 2012 at our department. For the purpose of comparability, the patient’s VMAT and IMRT plans were all carried out by one experienced dosimetrist in the area of head and neck IMRT planning. The distribution of clinical stages according to the American Joint Committee on Cancer (AJCC) Staging System 2010 was as follows: Stage I, 1 (5%); Stage II, 2 (10%); Stage III, 11 (55%); Stage IV, 6 (30%).

All patients were immobilized with thermoplastic mask. Computed Tomography (CT) (Siemens Sensation™, Munich, Germany) scan data (3 mm thickness, 512 × 512 pixels) was obtained from vertex to carina before imported into the Monaco 3.2 treatment planning system (TPS) (Elekta AB, Stockholm, Sweden).

In accordance with the Radiation Therapy Oncology Group (RTOG) 0225 and 0615 protocols [12,13], Gross Tumor Volume (GTV) covered gross tumor and regional metastatic nodes measuring more than 1 cm in diameter and/or showing central necrosis on the CT image. The CTV_72.6_ encompassed the GTV with a 0.5 cm margin; The CTV_63.6_, defined as the high-risk regions, included CTV_72.6_ and the entire nasopharynx, clivus, skull base, pterygoid fossae, parapharyngeal space, inferior sphenoid sinus, posterior third of the nasal cavity, maxillary sinuses as well as the retropharyngeal and upper deep jugular lymph node regions. Low jugular and supraclavicular regions with lymph node metastasis were also included in the CTV_63.6_. The CTV_54_, defined as the low-risk regions, included low jugular and supraclavicular regions without lymph node metastasis. The CTV_72.6_, CTV_63.6_ and CTV_54_ were all expanded by a 3 mm margin for PTV_72.6_, PTV_63.6_ and PTV_54_ to account for patient setup errors. The defined target volumes received three dose levels. The median absorbed-dose (D_50%_) prescriptions were 72.6 Gy/33f to PTV_72.6_, 63.6 Gy/33f to PTV_63.6_ and 54 Gy/28f to PTV_54_. According to ICRU83 [14], the planning objectives included: D_98%_ (near-min dose) of the PTV_72.6_, PTV_63.6_ and PTV_54_ are no less than 90% of planned absorbed dose, respectively. D_95%_ of the PTV_72.6_, PTV_63.6_ and PTV_54_ are no less than 95% of planned absorbed dose respectively. D_2%_ (near-max dose) of the PTV_72.6_ is no more than 107% of planned absorbed dose respectively. (D_2%_, D_98%_ and D_95%_ represent the minimum absorbed dose received by 2%, 98% and 95% of the target volume, respectively). Regarding the OARs, the maximum doses to the brain stem and the spinal cord were set as 54 Gy and 45 Gy, respectively. In addition, at least one of the parotid glands should receive a mean dose of no more than 26 Gy, or at least 50% of one gland should receive <30 Gy. The dose constraints to other normal tissues are all listed in Table 1.

**Table 1:** Dose constraints for the critical structures

**OARs**	**Dose constraints**
Brain stem	Max dose <54 Gy
Spinal cord	Max dose <45 Gy
Parotid glands	Mean dose < 26 Gy or V_30_ < 50% in one gland at least
Chiasm	Max dose <50 Gy or V_54_ < 1%
Optic nerves	Max dose <50 Gy or V_54_ < 1%
Lens	Max dose <10 Gy
Larynx	Mean dose < 45 Gy
Oral cavity	Mean dose <45 Gy

*Abbreviations*: *OARs*; Organs at risk, *Max dose*; Maximum dose, *Vx*; % volume receiving x Gy.

### Planning technique

VMAT and IMRT plans were all generated by Monaco 3.2 TPS on the Axesse™ linac. Coplanar beams using 6-MV photon were applied to all plans. On the Axesse™ linac, Integrity™ supports Continuous Variable Dose Rate (CVDR) which makes the delivery of the prescription faster and smoother when compared to Banned Variable Dose Rate (BVDR) [15]. CVDR of the Axesse™ linac ranges from 45 MU/min to 660 MU/min. The thansmission and penumbra measurements of newly designed MLC of the Agility™ have a significant improvement compared to previous published data [16]. The Agility™ has a160-leaf MLC of projected width 5 mm at the isocenter, designed to replace the tongue-and-groove system and allow for complete interdigitation and non-contiguous field shape. Instead of using a tongue-and-groove design to reduce interleaf leakage, the Agility™ slightly tilted the leaves relative to the actual beam divergence [17-19].And due to the dynamic jaws orthogonal to the direction of leaf movement, leaf transmission is no more than 0.5% [16,20]. Thanks to these designs, the Agility™ has avoided tongue-and-groove effect, which may be easily seen on RapidArc [21]. The maximum speed of the dynamic leaf guide (DLG) is 3 cm/s. MLC maximum speed is 3.5 cm/s and can approach 6.5 cm/s with the aid of the DLG. The gantry maximum rotation velocity is 6°/s. The minimum segment width was set at 5 mm with the minimum Monitor Units (MUs) of control points (CPs) at 1MU. The X-ray Voxel Monte Carlo (XVMC) algorithm is used for the final dose calculation and segment optimization [22], with a calculation grid of 3 mm and 3% standard deviation.

### dIMRT and ssIMRT

Both dIMRT and ssIMRT plans were generated with 9 equidistant coplanar beams uniformly distributed into 0°, 40°, 80°, 120°, 160°, 200°, 240°, 280°, 320°. The maximum CPs of each angle was 15.

### VMAT

The rotating arc was set from −180° to 180°. The maximum CPs for VMAT1 was 180, and the maximum CPs per arc for VMAT2 was 120, which has been shown to be adequate for both efficiency and plan quality in our department.

### Dose-volume histograms (DVHs) and dose comparisons

All the data is based on DVHs calculated using the Monaco 3.2 TPS (Elekta AB, Stockholm Sweden). The dosimetric comparison criteria were as follows:

1. Homogeneity Index (HI): used for evaluation of the PTV coverage by the prescription isodose. Formula: HI = (D_2%_-D_98%_)/D_50%_[23].

2. Conformity Index (CI): used for evaluation of the dose homogeneity within the PTV. Formula: CI = (TV_PV_ × TV_PV_)/(V_PTV_ × V_TV_). (V_TV_ is the treatment volume of the body receiving 95% of the prescribed dose, V_PTV_ is the volume of PTV, and TV_PV_ is the volume of V_PTV_ within the V_TV_). CI value will be less than one, and the closer the CI to one, the better the conformality [24].

3. Delivery efficiency and dose verification: MUs and control points per fraction and plan calculation time for all plans were recorded. Treatment time was measured from beam-on to beam-off including time for radiation delivery and gantry rotation but not time for patient set-up. The mean dose rate was derived by dividing the MUs by beam-on time. Dosimetric validation was performed for all plans before being transferred to the Axesse™ linac. The delivered dose was measured by a two-dimensional ionization chamber array MatriXX™ (IBA Dosimetry, Schwarzenbruck, Germany). The calculated doses and the measured doses were compared by way of the Omnipro I’mRT software (IBA Dosimetry, Schwarzenbruck, Germany) which employs the gamma evaluation criteria of 3% and 3 mm [25].

4. Organs at risk: the normal tissue doses of both VMAT and IMRT plans were calculated. In particular, the D_max_ (maximum point dose), D_2%_(near maximum dose) and D_mean_ (mean dose) to serial organs were determined, as well as the D_mean_ (mean dose) or D_x%_ (maximum dose encompassing x% of the OARs volume) to parallel organs.

### Statistical analysis

All analyses were performed using the SPSS version 17 statistical software (IBM SPSS Statistics). P-values are from two-sided tests. The normal distribution of variables was firstly checked with the Kolmogorov-Smirnov test. The differences in techniques were determined by the General Linear Model-univariate (GLM-u) procedure, and within-group differences between techniques were analyzed by post-hocmultiple comparisons LSD (Least-significant difference) method. *P* < 0.05 was considered statistically significant.

## Results

### PTV coverage

**Figure 1:**
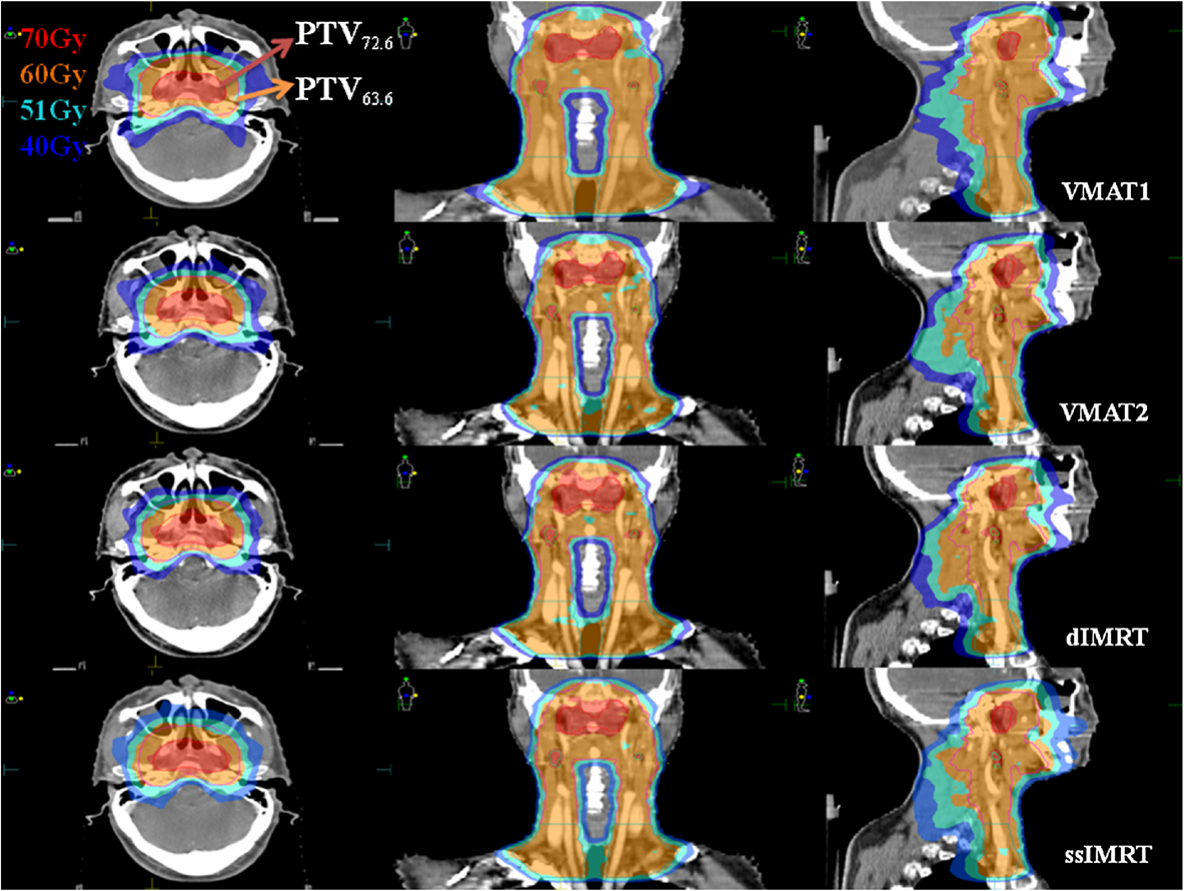
**The dose distributions for one NPC patient planned for VMAT1 (top), VMAT2 (second), dIMRT (third) and ssIMRT (bottom).** Color-wash areas: 70 Gy = red; 60 Gy = orange; 51 Gy = cyan; 40 Gy = blue; Red line is the outline of PTV_72.6_, wine and olive lines are the outlines of PTV_63.6_.

**Figure 2:**
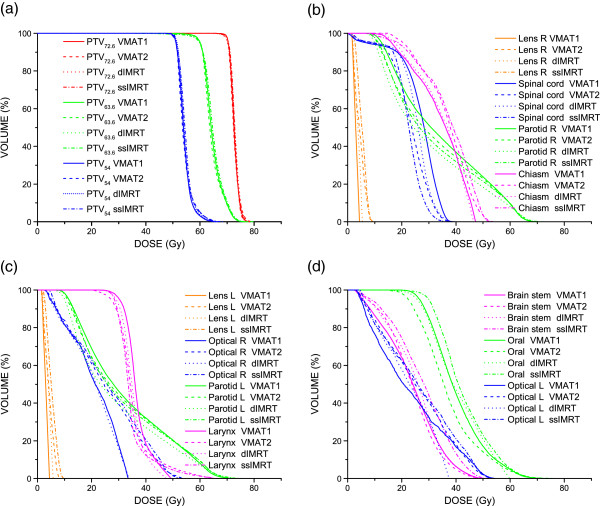
**The average DVH to the OARs of 20 NPC patients. (a)** The average DVH for all the PTVs comparing the four plan groups. **(b-d)** the average DVH to OARs of the four plan groups.

Dose distribution in all VMAT and dIMRT plans for all 20 patients satisfied clinical requirements. Typical dose distributions of VMAT1, VMAT2, dIMRT and ssIMRT planned for one NPC patient are shown in Figure 1. The average DVHs of the PTVs is shown in Figure 2a. The CI and HI of the PTVs are shown in Table 2. It is found that both VMAT1 and VMAT2 showed superior dose homogeneity and conformity in PTVs compared to dIMRT and ssIMRT. However, there was no significant difference in target coverage between VMAT1 and VMAT2.

**Table 2:** Comparison of target volume homogeneity and conformity in all four radiotherapy plan groups

		**VMAT1**	**VMAT2**	**dIMRT**	**ssIMRT**	***p***_**GLM-u**_	***p***_**VMAT1**_	***p***_**VMAT1**_	***p***_**VMAT1**_	***p***_**VMAT2**_	***p***_**VMAT2**_	***p***_**dIMRT**_
							_**-VMAT2**_	_**-dIMRT**_	_**-ssIMRT**_	_**-dIMRT**_	_**-ssIMRT**_	_**-ssIMRT**_
PTV_72.6_	HI	0.07 ± 0.01	0.07 ± 0.01	0.09 ± 0.01	0.09 ± 0.01	**<0.001**	0.510	**<0.001**	**<0.001**	**<0.001**	**<0.001**	0.653
	CI	0.57 ± 0.09	0.57 ± 0.08	0.49 ± 0.07	0.50 ± 0.07	**<0.001**	0.955	**<0.001**	**<0.001**	**<0.001**	**<0.001**	0.355
	D2%	75.43 ± 0.76	75.40 ± 0.89	76.30 ± 0.86	76.57 ± 0.66	0.472						
	D50%	72.64 ± 0.03	72.68 ± 0.03	72.93 ± 0.11	72.80 ± 0.10	0.588						
	D95%	70.55 ± 0.24	70.69 ± 0.30	70.18 ± 0.17	70.10 ± 0.23	0.464						
	D98%	70.01 ± 0.45	70.15 ± 0.57	70.01 ± 0.44	70.02 ± 0.40	0.742						
PTV_63.6_	HI	0.21 ± 0.02	0.21 ± 0.02	0.23 ± 0.02	0.23 ± 0.02	**<0.001**	0.427	**<0.001**	**<0.001**	**<0.001**	**<0.001**	0.614
	CI	0.76 ± 0.03	0.76 ± 0.04	0.73 ± 0.03	0.73 ± 0.04	**0.001**	0.448	**0.003**	**0.001**	**<0.001**	**<0.001**	0.605
	D2%	72.45 ± 1.11	72.42 ± 1.26	72.89 ± 1.16	73.20 ± 0.79	**0.005**	0.904	0.174	**0.003**	0.157	**0.002**	0.204
	D50%	64.14 ± 0.28	63.64 ± 0.11	63.99 ± 0.19	63.83 ± 0.17	0.236						
	D95%	60.42 ± 0.18	60.44 ± 0.12	60.20 ± 0.17	60.17 ± 0.27	0.715						
	D98%	58.49 ± 0.32	58.83 ± 0.39	57.99 ± 0.50	58.55 ± 0.41	**<0.001**	**0.007**	**<0.001**	0.651	**<0.001**	**0.021**	**<0.001**

HI: homogeneity index; CI: conformity index; GLM-u: general linear model-univariate; VMAT1: single arc VMAT, VMAT2: dual arc VMAT; dIMRT: dynamic MLC IMRT; ssIMRT: step-and-shoot IMRT. “Bold numbers” means that *p* value has a statistically significance.

### Dose to the OARs

The average dose and the maximum dose to the OARs for the 20 NPC patients are listed in Table 3. The average DVHs to the OARs for the 20 NPC patients are shown in Figure 2b-d. The four plan groups all meet well the requirements of the prescribed dose. The doses to normal tissues were within the clinically acceptable range. The four plan groups showed no significant difference in the doses to chiasm D_max_, left optic nerve D_max_ and right optic nerve D_mean_. In comparison with VMAT2, the D_mean_ of chiasm, left optic nerve and right lens in the VMAT1 plans was reduced, but the doses to spinal cord, oral cavity and larynx were increased. VMAT1 and VMAT2 showed no significant difference in normal tissue sparring. The dose to the right lens in the VMAT1 plans was reduced than that in the dIMRT and ssIMRT plans, while the doses to parotid gland and larynx in the VMAT1 plans were the opposite. The doses to oral cavity in the VMAT2 plans were lower than that in the dIMRT and ssIMRT plans, while VMAT2 plans had a higher dose to parotid gland compared to dIMRT plans.

**Table 3:** Dosimetric comparison of normal tissues in all four radiotherapy plan groups (Gy)

		**VMAT1**	**VMAT2**	**dIMRT**	**ssIMRT**	***p***_**GLM-u**_	***p***_**VMAT1**_	***p***_**VMAT1**_	***p***_**VMAT1**_	***p***_**VMAT2**_	***p***_**VMAT2**_	***p***_**dIMRT**_
							_**-VMAT2**_	_**-dIMRT**_	_**-ssIMRT**_	_**-dIMRT**_	_**-ssIMRT**_	_**-ssIMRT**_
Brain stem	D2%	41.21 ± 0.92	41.94 ± 0.42	42.56 ± 0.48	46.32 ± 0.44	**0.024**	0.679	0.444	**0.005**	0.724	**0.015**	**0.036**
	D_mean_	24.02 ± 2.58	23.46 ± 3.97	24.85 ± 3.86	27.51 ± 0.41	**<0.001**	0.326	0.056	**<0.001**	0.117	**<0.001**	**<0.001**
Spinal-cord	D2%	35.15 ± 1.75	32.59 ± 2.91	33.60 ± 2.67	28.66 ± 3.02	**<0.001**	**0.001**	0.073	**<0.001**	0.160	**<0.001**	**<0.001**
	D_mean_	26.26 ± 2.66	23.46 ± 2.74	25.01 ± 2.79	22.3 ± 3.52	**<0.001**	**<0.001**	0.055	**<0.001**	**0.017**	0.071	**0.001**
Chiasm	D_max_	47.51 ± 4.52	47.89 ± 5.01	47.17 ± 5.83	48.23 ± 6.32	0.600						
	D_mean_	34.44 ± 6.88	36.38 ± 5.79	35.30 ± 5.46	36.37 ± 7.30	**0.013**	**0.033**	0.427	**0.023**	0.330	0.988	0.337
Optic N L	D_max_	43.73 ± 10.41	46.20 ± 9.77	44.13 ± 9.52	45.11 ± 10.00	0.177						
	D_mean_	24.17 ± 9.69	26.17 ± 9.69	24.40 ± 8.29	26.22 ± 9.24	**0.022**	**0.021**	0.787	**0.018**	0.07	0.955	0.065
Optic N R	D_max_	43.30 ± 11.85	44.88 ± 9.57	42.04 ± 10.18	44.84 ± 8.91	**0.048**	0.177	0.278	0.188	**0.017**	0.971	**0.018**
	D_mean_	25.04 ± 9.69	25.66 ± 9.53	23.92 ± 8.74	26.04 ± 9.15	0.076						
Lens L	D_mean_	3.86 ± 1.35	4.13 ± 1.49	4.18 ± 1.38	5.18 ± 1.53	**<0.001**	0.111	0.06	**<0.001**	0.767	**<0.001**	**<0.001**
Lens R	D_mean_	3.71 ± 1.05	4.35 ± 1.7	4.21 ± 1.21	5.22 ± 1.38	**<0.001**	**0.004**	**0.022**	**<0.001**	0.535	**<0.001**	**<0.001**
Parotid L	D50%	28.45 ± 4.20	27.04 ± 4.99	23.89 ± 4.05	26.73 ± 4.09	**<0.001**	0.064	**<0.001**	**0.025**	**<0.001**	0.682	**<0.001**
	D_mean_	32.20 ± 3.30	31.57 ± 3.86	29.01 ± 3.31	31.48 ± 3.21	**<0.001**	0.223	**<0.001**	0.165	**<0.001**	0.863	**<0.001**
Parotid R	D50%	31.73 ± 5.88	28.18 ± 5.38	25.42 ± 5.53	27.07 ± 3.79	**<0.001**	**0.001**	**<0.001**	**<0.001**	**0.008**	0.273	0.104
	D_mean_	34.14 ± 3.56	32.47 ± 3.83	30.11 ± 3.93	31.65 ± 3.08	**<0.001**	0.085	**<0.001**	**0.001**	**0.001**	0.238	0.097
Oral cavity	D_mean_	40.35 ± 4.77	36.99 ± 6.18	41.42 ± 6.77	43.21 ± 5.60	**<0.001**	**<0.001**	0.230	**0.002**	**<0.001**	**<0.001**	**0.049**
Larynx	D_mean_	37.67 ± 4.13	35.17 ± 4.55	35.86 ± 3.05	34.76 ± 3.82	**<0.001**	**0.001**	**0.003**	**<0.001**	0.249	0.395	**0.048**

GLM-u: general linear model-univariate; VMAT1: single arc VMAT, VMAT2: dual arc VMAT; dIMRT: dynamic MLC IMRT; ssIMRT: step-and-shoot IMRT. Lens L, left lens; Lens R, right lens; Optic L, left optic nerve; Optic R, right optic nerve; Parotid L, left parotid; Parotid R, right parotid. “Bold numbers” means that *p* value has a statistically significance.

### Delivery efficiency and dose verification

The MUs, delivery time, mean dose rate, control points and plan calculation time of the four plan groups are all shown in Table 4. The MUs for the VMAT1, VMAT2, dIMRT and ssIMRT plans were 1232.1 ± 146.2, 1349.9 ± 133.8, 1292.2 ± 120.7 and 1090.2 ± 91.1, respectively. VMAT1 had lower MUs than VMAT2, and the MUs of ssIMRT Significantly reduced compared to both of VMAT. Delivery time for the VMAT1, VMAT2, dIMRT and ssIMRT plans were 2.7 ± 0.2 min, 3.9 ± 0.3 min, 5.7 ± 0.2 min and 14.1 ± 1.0 min, respectively. VMAT1 reduced the average delivery time by 29.8%, 51.1% and 80.8% compared with VMAT2, dIMRT and ssIMRT, respectively. VMAT2 reduced the average delivery time by 30.2% and 72.3% compared with dIMRT and ssIMRT. The mean dose rates for the VMAT1, VMAT2, dIMRT and ssIMRT plans were 494.0 ± 38.6 MU/min, 379.6 ± 30.1 MU/min, 249.8 ± 17.9 MU/min and 86.6 ± 5.1 MU/min, respectively. VMAT1 and VMAT2 increased the mean dose rate by 97.7% and 51.9% in comparison with dIMRT, and by 470.4% and 338.3% in comparison with ssIMRT. Dosimetric verification showed that all four techniques were accompanied by a high quality assurance. Average γ pass rates for VMAT1, VMAT2, dIMRT and ssIMRT using the 3 mm/3% gamma criteria were 96.3%, 95.4%, 96.5% and 96.2%, respectively.

**Table 4:** Comparison of delivery efficiency in all four radiotherapy plan groups

	**VMAT1**	**VMAT2**	**dIMRT**	**ssIMRT**	***p***_**GLM-u**_	***p***_**VMAT1 -VMAT2**_	***p***_**VMAT1 -dIMRT**_	***p***_**VMAT1 -ssIMRT**_	***p***_**VMAT2 -dIMRT**_	***p***_**VMAT2 -ssIMRT**_	***p***_**dIMRT -ssIMRT**_
MUs	1232.1 ± 146.2	1349.9 ± 133.8	1292.2 ± 120.7	1090.2 ± 91.1	**<0.001**	**0.001**	0.067	**<0.001**	0.079	**<0.001**	**<0.001**
Time (min)	2.7 ± 0.2	3.9 ± 0.3	5.7 ± 0.2	14.1 ± 1.0	**<0.001**	**<0.001**	**<0.001**	**<0.001**	**<0.001**	**<0.001**	**<0.001**
Mean dose rate (MU/min)	494.0 ± 38.6	379.6 ± 30.1	249.8 ± 17.9	86.6 ± 5.1	**<0.001**	**<0.001**	**<0.001**	**<0.001**	**<0.001**	**<0.001**	**<0.001**
Control points	161.4 ± 13.1	187.0 ± 30.9	126.6 ± 10.2	138.0 ± 10.4	**<0.001**	**<0.001**	**<0.001**	**<0.001**	**<0.001**	**<0.001**	0.223
plan calculation time (hour)	5.6 ± 1.4	4.8 ± 1.2	0.8 ± 0.3	0.8 ± 0.4	**<0.001**	0.092	**<0.001**	**<0.001**	**<0.001**	**<0.001**	0.945

GLM-u: general linear model-univariate; VMAT1: single arc VMAT, VMAT2: dual arc VMAT; dIMRT: dynamic MLC IMRT; ssIMRT: step-and-shoot IMRT. “Bold numbers” means that *p* value has a statistically significance.

## Discussion

Target volume conformity and homogeneity are closely related to the complexity of target volumes, the delineation of volumes, the delivery equipment, the radiotherapy technology and the optimization algorithm. Single arc VMAT has been shown to successfully meet the clinical requirements of intensity modulation radiotherapy for simple target volume. For example, a recent study concluded that single-arc VMAT for prostate cancer was dosimetrically equivalent to fixed-beam IMRT [26]. Most previous studies have indicated that, for complex target volumes such as those seen in head and neck cancer, single-arc VMAT may be less favorable dosimetrically than a fixed field IMRT [11,27,28]. This study has shown that target volume coverage and normal tissue sparing with VMAT1 is not significantly different from VMAT2. As expected, the target volume conformity and homogeneity of VMRT1 are better than that of IMRT.

In the past, it has been difficult to optimize single-arc VMAT for complex target volumes in head and neck cancer, especially NPC. When the optimization of a certain segment cannot meet the requirements, the TPS can rely on another arc to make up for the lack of intensity modulation. When single-arc VMAT was used in the treatment of NPC, smaller and faster MLCs, as well as the CVDR were sometimes needed to meet the plan optimization requirements. We noted that 10 mm width and 2 cm s^-1^ velocity MLCs without interdigitation and BVDR were mainly used in the previous studies [11,27,28]. Restricted by the mechanical components of linacs and MLCs, single-arc VMAT in these studies could not fully meet the intensity modulation requirements for the complex target volumes, therefore dosimetric distributions were inferior to double arc VMAT and IMRT. It has been reported that the 5 mm MLC width generates better treatment plans than the 10 mm MLC [29]. The Axesse™ linac can deliver CVDR and has much smaller and faster interdigitated MLCs, thus generating high quality plans by improving optimization significantly. Single-arc VMAT dosimetric distribution in this study was tailored to allow complex dose shaping around the NPC target volume. The results of this study has shown that single-arc VMAT, when applied to a complex target volume such as NPC using the Axesse™ linac, is superior to IMRT and not inferior to VMAT2 for target coverage. And VMAT1 shortened delivery time and significantly improved delivery efficiency compared to VMAT2 and IMRT.

Previous results reported by Lu et al. [30] and White et al. [31] showed that double-arc VMAT for NPC achieved significant improvements in dose reduction to OARs and healthy tissue sparing compared with IMRT. On the other hand, Kan et al. [32] indicated that double-arc VMAT produced slightly inferior parotid sparing than nine-field “sliding-window” IMRT. A similar result was reported by Zhang et al. [28], who showed that the dose distribution of single-arc VMAT plan for NPC was slightly worse than that of a nine-field IMRT plan. In this study, we found that VMAT had improved plan quality in terms of target coverage compared to IMRT, but had no obvious advantage over IMRT in normal tissue sparing, even dIMRT showed an improved sparing of the parotid gland. The reported inconsistency might be related to the complexity of the target volume, the contouring of OARs and the dose constraints set. It is therefore difficult to draw a definite conclusion on single-arc VMAT for all NPC cases.

Similar to the previous VMAT studies [11,26,28], the delivery efficiency of VMAT in this study, especially VMAT1, was significantly superior to dIMRT and ssIMRT. VMAT1 reduced the average treatment time by 80.8%, 51.1% and 29.8% respectively compared with ssIMRT, dIMRT and VMAT2. When VMAT is applied in the treatment of NPC, delivery efficiency is related to the number of arcs, the dose rate and the MLC movement velocity. In vitro and in vivo experiments have shown that the prolonged delivery time shortened the tumor growth delay and survival time in tumor-bearing mice and the radiobiological effect decreased with the elongation of treatment time [33,34]. A shorter delivery time with VMAT1 could theoretically improve the radiobiological effect, however the clinical outcomes need further verification. As reported by Bertelsen et al. [35] and Cao et al. [15], the use of CVDR for VMAT reduced significantly the treatment time compared to BVDR. At the same time, it has been shown that beam-on time would decrease 10 to 40 seconds for every 100 MU/min increase of dose rate [36]. Therefore this study has shown that the optimized single-arc VMAT plan by using continuously variable high dose rate (mean dose rate 494 MU/min), can improve delivery efficiency.

It has been reported that the MUs in VMAT plans for NPC cases were significantly lower than in IMRT plans [11,32]. In our study, VMAT plans improved delivery efficiency but did not significantly reduce MUs, compared with IMRT plans. To the authors’ knowledge, the number of MUs for a given NPC plan is related to the adopted radiotherapy technique, TPS and the mechanical characteristics of the accelerator and MLCs.In comparison with other VMAT plans for NPC using the Monaco TPS [28], the number of VMAT MUs in this study was high for two reasons. Firstly, the optimized VMAT plan used a higher dose rate. The number of MUs increases with a higher dose rate, but the influence of a higher dose rate on the number of MUs decreases significantly with the increase of MLC velocity [37]. Similar reports have shown that a higher dose rate will increase the number of MUs and reduce the beam-on time in IMRT [36]. We can see in this study that the effect of a higher dose rate on the MUs didn’t reduce the delivery efficiency of the VMAT plans. Secondly, the other reason for the increased MUs noted in VMAT plans is due to the interdigitation ability of the MLCs as well as threshold of the minimum segment width (5 mm) and the minimum CPs MU (1 MU). To be more specific, the optimized VMAT plans in this study generated a greater number of smaller and narrower segments, with less than 5 MUs in 20 percent of the CPs. Although this helped improve plan quality, it also increased the number of MUs.

## Conclusions

Based on an Axesse™ linear accelerator, single-arc VMAT for the treatment of NPC is entirely feasible, and furthermore provides better delivery efficiency than dual-arc VMAT, dIMRT, and ssIMRT. Similar PTV coverage and sparing of OARs were observed in both VMAT delivery techniques. And both single-arc VMAT and dual-arc VMAT had improved target coverage compared to IMRT, but there is still room for improvement in terms of OAR sparing, such as parotid gland. The impact of reduced delivery time on clinical outcomes needs further investigation.

## Competing interests

None of the authors report any competing interests.

## Authors’ contributions

HLP is responsible for the conception and study design. ZHN, WDG, JH, YH and HLP collected the data and contributed the cases. ZHN created the manuscript draft. JXJ, QLL and JMM supervised the medical physics. All authors read and approved the final manuscript.
